# Characterization of virulent Newcastle disease viruses from vaccinated chicken flocks in Eastern China

**DOI:** 10.1186/s12917-016-0732-6

**Published:** 2016-06-16

**Authors:** Jie Zhu, Shunlin Hu, Haixu Xu, Jingjing Liu, Zhenzhen Zhao, Xiaoquan Wang, Xiufan Liu

**Affiliations:** Animal Infectious Disease Laboratory, College of Veterinary Medicine, Yangzhou University, 12 East Wenhui Road, Yangzhou, Jiangsu 225009 China; Jiangsu Co-innovation Center for Prevention and Control of Important Animal Infectious Diseases and Zoonoses, Yangzhou University, Yangzhou, 225009 China; Shandong Binzhou Wohua Biological Engineering Co., Ltd, Binzhou, 256600 China

**Keywords:** Virulent Newcastle disease virus, Sub-genotype VIId, E347K, Variation, Antigenic difference

## Abstract

**Background:**

Newcastle disease (ND) is one of the most contagious and devastating diseases to poultry in the world. The causative agents are virulent strains of Newcastle disease virus (NDV), which belong to the genus Avulavirus, sub-family Paramyxoviridae, family Paramyxovirinae. Knowing the genomic and antigenic characteristics of virulent NDVs might contribute to ND control in China.

**Results:**

The results showed that all of the virulent strains belonged sub-genotype VIId shared the same cleavage site ^112^RRQKR/F^117^ in the fusion protein. At least 69 % (38 of 55) of the NDV strains possessed E347K variation in the hemagglutinin-neuraminidase protein. The cross-neutralization tests confirmed that the strains harboring 347 K showed lower antigenic relatedness with LaSota. Furthermore, the immune-challenge experiment indicated that LaSota could not provide complete protection against infection with the E347K variant NDVs as the vaccinated birds were still able to be infected and shed virulent challenge viruses.

**Conclusions:**

Currently, sub-genotype VIId NDVs are the prevalent virulent strains circulating among vaccinated chicken flocks in Eastern China. Our findings indicated that the E347K variation in HN gene would expand the antigenic difference with LaSota, which may be responsible for the increasing isolation rate of these strains from vaccinated chickens.

## Background

Newcastle disease (ND) is one of the most contagious and devastating diseases to poultry in the world [1]. The causative agents are virulent strains of Newcastle disease virus (NDV), which belong to the genus Avulavirus, sub-family Paramyxoviridae, family Paramyxovirinae [2, 3]. The viral RNA genome is approximately 15 kb in length and encodes six major virus proteins: the nucleocapsid protein (NP), phosphoprotein (P), matrix protein (M), fusion protein (F), hemagglutinin–neuraminidase (HN) and large polymerase protein (L). In addition, the HN protein is an important multifunctional surface glycoprotein that consists of a cytoplasmic domain, a transmembrane region, a stalk region and a globular head, in which the receptor–binding site, the site responsible for neuraminidase activity and all of the antigenic sites reside [4]. It has been confirmed that there are seven overlapping antigenic sites in the HN protein, whereas residues 345 to 353 constitute the only linear epitope identified in the HN gene, which is susceptible to immune pressure to generate antigenic variation [5]. Researchers have recently confirmed that the variation in the linear epitope in the HN protein would intensify the antigenic difference [6]. Previously, seven neutralizing epitopes positioned at residues 72, 74, 75, 78, 79, 157 to 171, and 343 of the F protein have been identified.

The strains of NDV can be divided into two distinct clades: class I and class II, and based on the older classification system, both clades could be divided into 9 genotypes, and the class II clade can be divided into fifteen genotypes when based on the new classification system, while the class I clade can be divided into three sub-genotypes. To date, a strict vaccination policy has been implemented in China and the vaccine strain LaSota which belonged to genotype II has been widely used in China for over 40 years. However, infections of genotype VIId NDVs have still frequently occurred in China and other Asian countries [7–13]. Meanwhile, there are also some virulent genotypes (VIb, IX) circulating among avian species in China [14, 15].

To elucidate the circulation of virulent NDVs among chickens in Eastern China, 55 virulent NDVs isolated from chicken flocks, including broilers and layers, from 2011 to 2013 were phylogenetically characterized.

## Results

### Isolation and identification of NDVs from clinical samples

Fifty-five NDVs from clinical samples of different chicken flocks in Eastern China were isolated from 2011 to 2013, identified and plaque-purified, and the details of the NDV isolates are shown in Table 1.

**Table 1:** NDVs characterized in this study

NDV isolates	Year of isolation	Host	F gene	345–353 Residues of HN	HN gene	NDV isolates	Year of isolation	Host	F gene	345–353 Residues of HN	HN gene
JS-01-11-Ch	2011	Broiler	JQ013855	PDEQDYQIR	JQ013835	JS-09-12-Ch	2012	Layer	KJ184579	PDKQDYQIR	KJ184625
JS-03-11-Ch	2011	Broiler	JQ013857	PDEQDYQIR	JQ013838	JS-10-12-Ch	2012	Layer	KJ184580	PDKQDYQIR	KJ184626
JS-04-11-Ch	2011	Layer	JQ013858	PDEQDYQIR	KJ184630	JS-11-12- Ch	2012	Layer	KJ184581	PDKQDYQIR	KJ184627
JS-05-11-Ch	2011	Layer	JQ013859	PDEQDYQIR	KJ184631	JS-12-12-Ch	2012	Broiler	KJ184582	PDKQDYQIR	KJ184601
JS-06-11-Ch	2011	Layer	JQ013860	PDKQDYQIR	JQ013839	JS-13-12-Ch	2012	Broiler	KJ184583	PDKQDYQIR	KJ184602
JS-07-11-Ch	2011	Broiler	JQ013861	PDKQDYQIR	KJ184632	JS-14-12-Ch	2012	Broiler	KJ184584	PDKQDYQIR	KJ184603
JS-08-11-Ch	2011	Broiler	JQ013862	PDGQDYQIR	KJ184633	AH-02-12-Ch	2012	Layer	KJ184597	PDKQDYQIR	KJ184616
JS-09-11-Ch	2011	Broiler	JQ013863	PDEQDYQIR	KJ184634	HeN-01-12-Ch	2012	Layer	KJ184598	PDKQDYQIR	KJ184617
JS-10-11-Ch	2011	Layer	JQ013864	PDGQDYQIR	JQ013840	SD-01-12-Ch	2012	Broiler	KJ184594	PDKQDYQIR	KJ184613
JS-11-11-Ch	2011	Layer	JQ013865	PDKQDYQIR	JQ013841	SD-02-12-Ch	2012	Broiler	KJ184595	PDKQDYQIR	KJ184614
JS-12-11-Ch	2011	Broiler	JQ013866	PDEQDYQIR	JQ013842	SD-03-12-Ch	2012	Broiler	KJ184596	PDKQDYQIR	KJ184615
JS-13-11-Ch	2011	Broiler	JQ013867	PDEQDYQIR	JQ013843	JS-16-12-Ch	2012	Broiler	KJ184585	PDKQDYQIR	KJ184604
JS-14-11-Ch	2011	Broiler	JQ013868	PDKQDYQIR	JQ013852	JS-17-12-Ch	2012	Broiler	KJ184586	PDKQDYQIR	KJ184605
JS-15-11-Ch	2011	Broiler	JQ013869	PDEQDYQIR	JQ013844	JS-18-12- Ch	2012	Broiler	KJ184587	PDEQDYQIR	KJ184606
JS-16-11-Ch	2011	Broiler	JQ013870	PDKQDYQIR	JQ013845	JS-19-12-Ch	2012	Broiler	KJ184588	PDKQDYQIR	KJ184607
JS-17-11-Ch	2011	Layer	JQ013871	PDKQDYQIR	JQ013846	JS-20-12-Ch	2012	Broiler	KJ184589	PDKQDYQIR	KJ184608
JS-19-11-Ch	2011	Layer	JQ013873	PDKQDYQIR	JQ013847	JS-21-12-Ch	2012	Broiler	KJ184590	PDKQDYQIR	KJ184609
JS-20-11-Ch	2011	Broiler	JQ013874	PDEQDYQIR	JQ013848	JS-22-12-Ch	2012	Broiler	KJ184591	PDEQDYQIR	KJ184610
JS-21-11-Ch	2011	Broiler	JQ013875	PDEQDYQIR	JQ013851	JS-23-12-Ch	2012	Broiler	KJ184592	PDKQDYQIR	KJ184611
SD-01-11-Ch	2011	Layer	JQ013877	PDKQDYQIR	JQ013850	JS-24-12-Ch	2012	Broiler	KJ184593	PDKQDYQIR	KJ184612
SD-02-11-Ch	2011	Layer	JQ013878	PDKQDYQIR	JQ013849	JS-21-13-Ch	2013	Layer	KP064014	PDKQDYQIR	KP064023
JS-02-12-Ch	2012	Layer	KJ184599	PDKQDYQIR	KJ184618	JS-22-13-Ch	2013	Broiler	KP064015	PDKQDYQIR	KP064024
JS-03-12-Ch	2012	Broiler	KJ184600	PDEQDYQIR	KJ184619	JS-23-13-Ch	2013	Broiler	KP064016	PDKQDYQIR	KP064025
JS-04-12-Ch	2012	Broiler	KJ184574	PDEQDYQIR	KJ184620	JS-24-13-Ch	2013	Layer	KP064017	PDKQDYQIR	KP064027
JS-05-12-Ch	2012	Broiler	KJ184575	PDEQDYQIR	KJ184621	JS-27-13-Ch	2013	Layer	KP064018	PDKQDYQIR	KP064021
JS-06-12-Ch	2012	Layer	KJ184576	PDKQDYQIR	KJ184622	JS-30-13-Ch	2013	Broiler	KP064020	PDKQDYQIR	KP064026
JS-07-12-Ch	2012	Layer	KJ184577	PDKQDYQIR	KJ184623	SD-25-13-Ch	2013	Broiler	KP064019	PDKQDYQIR	KP064022
JS-08-12-Ch	2012	Broiler	KJ184578	PDKQDYQIR	KJ184624						

### Phylogenetic analysis

**Fig. 1:**
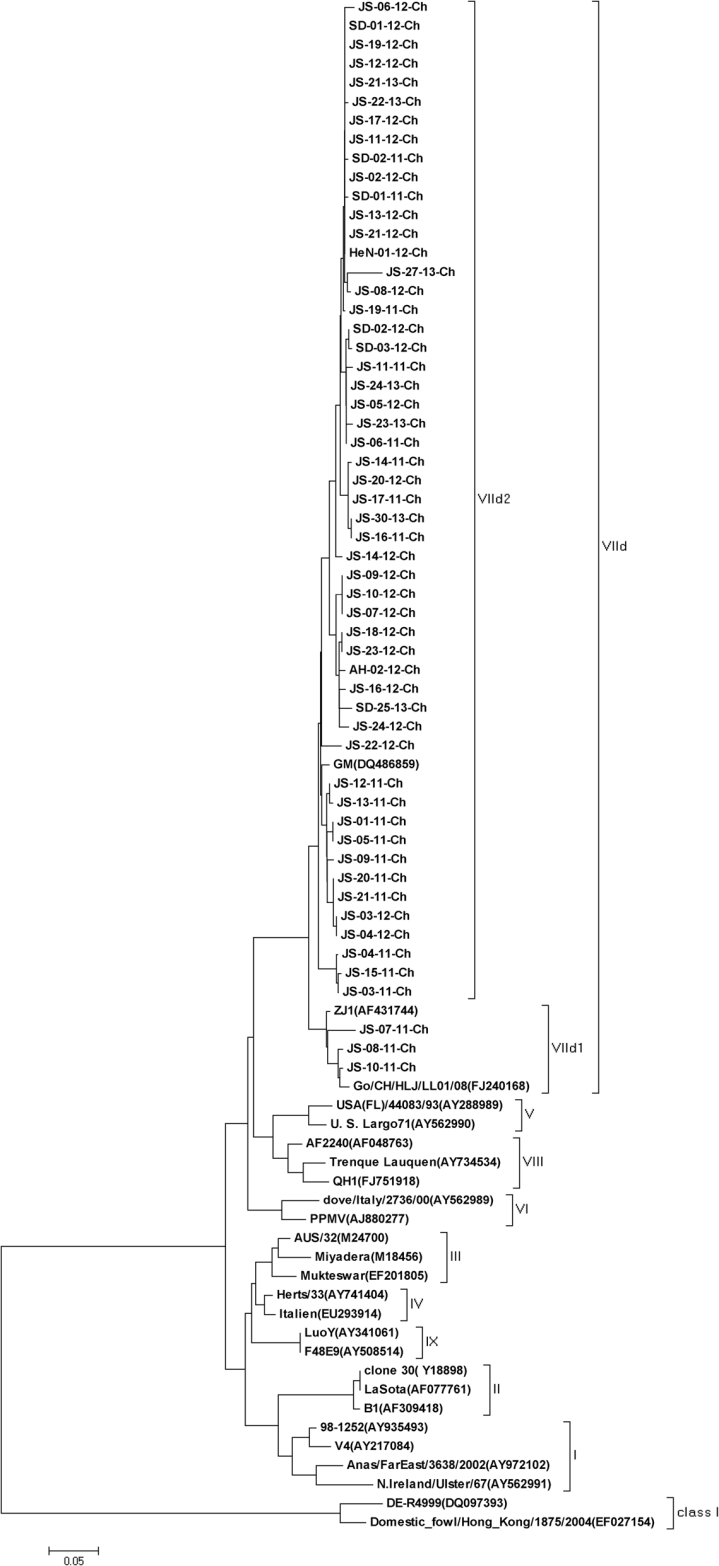
Phylogenetic tree of 55 sub-genotype VIId NDV strains based on the whole region of the F gene

The phylogenetic analysis of the 55 NDVs based on the whole F gene sequences showed that all strains were clustered into sub-genotype VIId and they could be divided into two separate groups, namely VIId1 (*n* = 3) and VIId2 (*n* = 52), as previously described (Fig. 1) [7]. All of the strains isolated in 2012–2013 belonged to VIId2, and only three of the twenty one strains isolated in 2011 belonged to VIId1. All of the NDV isolates possessed the virulent F protein cleavage site motif ^112^RRQKR/F^117^. And over 70 % (39/55) of the isolates shared K78R variation on the F gene.

**Fig. 2:**
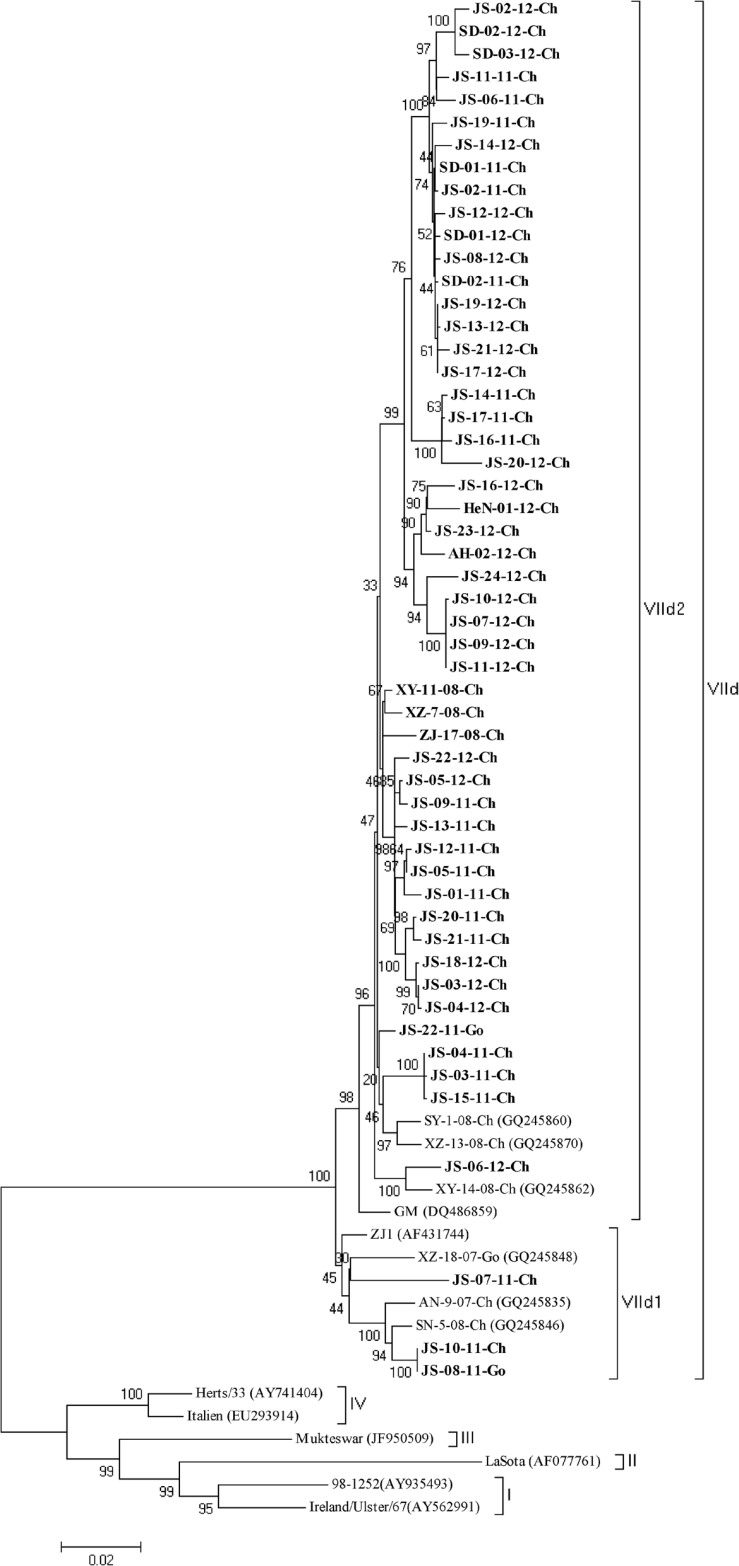
Phylogenetic tree of 55 sub-genotype VIId NDV strains based on the complete HN gene

Additionally, a phylogenetic analysis was also performed based on the whole HN gene (Fig. 2). As determined from the phylogenetic tree based on the HN gene, all of the NDV isolates showed a distribution similar to that obtained from the tree based on the F genes: three in VIId1 and 52 in VIId2. Three residues, namely T102, A118 and T443, were unique for VIId1, whereas I102, E118 and M443 were characteristic residues of VIId2. All of the NDV isolates shared 96.3 % to 100 % nucleotide identity and 96.2 % to 99.8 % amino acid identity. Only two isolates (JS-08-11-Ch and JS-10-11-Ch) shared the E347G variation. More than 42.8 % of the isolates in 2011 (9/21) exhibited the E347K variation in HN, whereas 81.5 % (22 of 27) of those isolated in 2012 possessed this variation. The latter percentage is nearly two-fold higher than that found in 2011, and 100 % (7 of 7) of the strains isolated in 2013 possessed this variation. In addition, the layer-origin stains isolated in 2011, 2012 and 2013 shared 67 % (6/9), 75 % (6/8) and 100 % (3/3) of the E347K variation in the HN gene, respectively. Furthermore, we found that all of the strains harboring 347 K on HN also shared a G362A mutation. However, the role of this mutation has not been well elucidated. The 13 cysteine residues were conserved as described previously, and with the exception of the loss of a glycosylation site at 508 of JS-02-11, all of the other NDVs possessed six well-identified glycosylation sites.

### Pathogenicity tests

As is shown in Table 2, all the ten isolates were highly virulent, which were consistent with the tyipical virulent motif ^112^RRQKRF^117^ at the F cleavage sites.

**Table 2:** Coefficients of antigenic similarity (R) between NDV isolates and LaSota strain

Strains	*R* value^c^	345–353 Residues of HN
SD-01-11-Ch	0.04^a^ 0.12^b^	PD**K**QDYQIR
JS-07-11-Ch	0.12^a^ 0.12^b^	PD**K**QDYQIR
JS-14-12-Ch	0.06^a^ 0.12^b^	PD**K**QDYQIR
HeN-01-12-Ch	0.12^a^ 0.12^b^	PD**K**QDYQIR
JS-27-13-Ch	0.17^a^ 0.25^b^	PD**K**QDYQIR
JS-08-11-Ch	0.35^a^ 0.35^b^	PD**G**QDYQIR
JS-15-11-Ch	0.35^a^ 0.5^b^	PD**E**QDYQIR
JS-05-12-Ch	0.35^a^ 0.35^b^	PD**E**QDYQIR
JS-18-12-Ch	0.25^a^ 0.35^b^	PD**E**QDYQIR
JS-22-12-Ch	0.35^a^ 0.35^b^	PD**E**QDYQIR

^a^Chicken embryo cross-neutralization test

^b^Cross-hemagglutination inhibition test

^c^0.67 ≤ *R ≤* 1.5, indicates no significant antigenic difference between the two viruses

0.5 ≤ *R* ≤ 0.67 indicates a minor difference between the two viruses

*R* < 0.5 indicates a major difference between the two virus strains

### Cross hemagglutination inhibition (HI) test and virus neutralization test

The HI titers of the anti-LaSota serum for the ten isolates were 4-8-fold lower than that for the homologous strain LaSota, displaying the significance of the antigenic difference between the NDV isolates and the vaccine strain LaSota. As determined from the cross HI test, the R values between the predominant strains and LaSota were all lower than 0.5, indicating the existence of a significant antigenic difference between the strains. Meanwhile the R values between the E347K-variant strains and LaSota were lower than that found for the 347E/G strains, which indicate that the E/G347K variation would expand the antigenic difference with the vaccine strain. And the significant antigenic difference was also confirmed by the R value obtained from cross-neutralization test between LaSota and the predominant strains (Table 3).

**Table 3:** Pathogenicity tests of the isolates

Strains	ICPI^a^	MDT^b^ (h)
SD-01-11-Ch	1.84	48
JS-07-11-Ch	1.86	46.8
JS-14-12-Ch	1.94	43.8
HeN-01-12-Ch	1.82	52
JS-27-13-Ch	1.86	46
JS-08-11-Ch	1.94	46
JS-15-11-Ch	1.92	48
JS-05-12-Ch	1.96	49.2
JS-18-12-Ch	1.84	48
JS-22-12-Ch	1.86	46

^a^MDT = mean death time

^b^ICPI = intracerebral pathogenicity index

### Test of the protective efficacy of the LaSota strain against the variant strains

As is shown in Table 4, none of the birds vaccinated with the LaSota vaccine showed clinical signs post challenge with JS-22-11-Ch or JS-14-12-Ch. In contrast, 100 % of the unvaccinated birds challenged with JS-22-11-Ch or JS-14-12-Ch died within five days post challenge. All of the unchallenged birds remained normal throughout the experiment.

**Table 4:** Protection of vaccinated SPF chickens with inactivated oil-emulsion vaccines against challenge with the predominant variant NDVs

Vaccine-Challenge^a^	HI titer(log2) Prior to challenge	Challenge			Protection	
		Strain	Route	NO. birds	Clinical signs	Mortality
La-JS22	6.3 ± 1.8	JS-01-11-Ch	ED/IN	10	0/10	0 % (0/10)
La-JS14	6.3 ± 1.8	JS-14-12-Ch	ED/IN	10	0/10	0 % (0/10)
Control-JS22	0	JS-01-11-Ch	ED/IN	5	5/5	100 % (5/5)
Control –JS14	0	JS-14-12-Ch	ED/IN	5	5/5	100 % (5/5)
La-PBS	6.3 ± 1.8	PBS	ED/IN	5	0/10	0 % (0/5)
Control-PBS	0	PBS	ED/IN	5	0/10	0 % (0/5)

^a^La-JS22 and Control-JS22 = Challenge with 10^6^ EID_50_ JS-22-11-Ch; La-JS14 and Control-JS14 = Challenge with 10^6^ EID_50_ JS-14-12-Ch

As is shown in Table 5, tracheal and cloacal swabs were collected from the birds at days 3, 5, and 7 post challenge and subjected to virus isolation tests. The virus isolation rate from the tracheal swabs was 100 % (5/5) at day 3 post challenge in both the control-JS22 and control-JS14 groups, whereas the isolation rates for the La-JS22 and La-JS14 groups were 70 % (7/10) and 100 % (10/10), respectively. And the virus isolation rate from the tracheal swabs was 50 % (5/10) and 70 % (7/10) at day 5 post challenge in La-JS22 and La-JS14 groups, whereas the isolation rates at day 7 post challenge were 10 % (1/10) and 30 % (3/10), respectively.

**Table 5:** Frequency of isolation of challenge virus from SPF chickens

Group	Samples (positive/total)					
	Day 3 pc		Day 5 pc		Day 7 pc	
	OS	CS	OS	CS	OS	CS
La-JS22	7/10	2/10	5/10	6/10	1/10	2/10
La-JS14	10/10	5/10	7/10	7/10	3/10	4/10
Control-SD	5/5	5/5	–	–	–	–
Control-JS	5/5	5/5	–	–	–	–

–the birds were all dead within the day 5pc

OS = oral swabs; CS = cloacal swabs

## Discussion

The genotype VII NDVs have become the most prevalent strains in China since the 1990s, and the sub-genotype VIId NDVs are mainly responsible for the present ND epizootic in China [7, 13, 16]. In this study, virulent NDVs from the vaccinated chicken flocks were characterized as sub-genotype VIId and could be clustered into sub-genotype VIId1 and VIId2 based on the phylogenetic trees based on both F and HN genes [7]. T102, A118, and T443 of the HN gene were unique residue substitutions found in the VIId1 NDVs, and I102, E118, and M443 of the HN gene were characteristic residues of the VIId2 NDVs. In this study, we found that sub-genotype VIId2 remains the predominant genotype VII NDVs from 2011 to 2013, and all of the NDVs isolated from 2012 to 2013 were located in this clade.

The HN protein of NDV plays an important role in immune protection against virus infection, and the variation of the antigenic epitope would expand the antigenic difference. And analysis of the role of site 347 in antigenicity has confirmed that the E347K variation would intensify the antigenic difference and may increase the risk of vaccine breakdown [17, 18]. The isolation rate of strains with the E347K variation is increasing yearly, as previous reported. More than 36 % of the virus strains (20/55) were isolated in commercial layer flocks, which have resulted in losses in egg production. Cho et al. [18] found that the Korean variation strains have also caused losses in egg production. Moreover, more than 75 % (15/20) of the NDV strains isolated from layer flocks were the E347K-variation strains. The high isolation rate indicates that the E347K-variation strains can infect chickens under immune pressure because the layers were all well-vaccinated in China. Both site 347 and site 362 locate around the receptor-binding pocket, and we found of E347K, G362A co-variant strains, whereas Cho et al. also identified two strains with the E347K and G362R co-variant ones. However, the role of the co-variation remains unknown [6].

At present, LaSota is widely and frequently used to prevent NDV infection in the poultry industries in China; however, virulent NDV infections continue to occur in the well-vaccinated chicken flocks [12, 19]. Moreover, both cross HI and cross-neutralization tests were performed, and the R values of all of the strains were lower than 0.5. However, the R values between the E347K-variant strains and LaSota were lower than that found for the 347E/G strains, which indicate that the E347K variation would expand the antigenic difference with the vaccine strain. Therefore, the results of the cross-neutralization and cross HI experiments may also be consistent with the higher isolation rate of the E347K-variant strains, which demonstrated significant antigenic differences between the vaccine strain and the predominant variant strains should be responsible for the outbreaks of ND in China.

In addition, the protection efficiency of LaSota against the E347K variation strains in China is not well-elucidated. In this study, the vaccinated SPF chickens were fully protected against morbidity and mortality, but virus shedding was not stopped. Our data suggest that the LaSota inactivated vaccine cannot protect chickens from virus shedding when infected with the variant NDVs, and the variant strain challenged group showed higher virus isoation rate which may be one of the reasons for the circulation of sub-genotype VIId variation strains in vaccinated chicken flocks.

In conclusion, our findings indicate that the sub-genotype VIId NDVs with E347K variation in the HN protein are predominant in eastern China and that this variation would intensify the antigenic difference with the vaccine strains. Moreover, the vaccine strain LaSota has been used for more than 40 years and can hardly protect chickens effectively from virus shedding when infected with the circulation strains. Therefore, new NDV vaccines closely related to the prevalent genotype VIId viruses should be developed to control ND in China.

## Conclusions

Currently, sub-genotype VIId NDVs are the prevalent virulent strains circulating among vaccinated chicken flocks in Eastern China. Our findings indicate that the E347K variation in HN protein would expand the antigenic difference with LaSota, which may be responsible for the increasing isolation rate of these strains from vaccinated chickens.

## Methods

### Virus isolation and antiserum preparation

Fifty-five virulent NDVs were isolated from 190 clinical samples from different flocks in Eastern China from 2011 to 2013 under the permission of the flock owners. All of the viruses were plaque-purified three times on primary chicken embryo fibroblasts (CEF) and proliferated in 9-10-day-old specific-pathogen-free (SPF) chicken embryos [20]. The virus stocks grown in allantoic fluids were stored at −70 °C until use.

SPF chickens (five in each group) were vaccinated with inactivated, oil-emulsion derived from LaSota and the strains listed in Table 2. The serum for the five birds vaccinated with the same vaccine were pooled together at three weeks post-vaccination and stored at −70 °C until use.

All of the SPF chicken embryos and the SPF chickens used in this study were bought from Beijing Merial Vital Laboratory Technology Co., LTD.

### Viral RNA purification, RT-PCR, and sequencing of the F and HN genes

Virus preparation, viral RNA extraction and the initial RT reaction were performed as described previously [21]. Based on the published F and HN gene sequences in the GenBank database, we designed two pairs of primers (Table 6) for amplifying the F and HN genes of the virulent NDV isolates. And we also designed a pair of primers based one the same region of all NDV F genes which could be used for NDV identification. PCR products possessing the expected length were purified and sequenced by Sangon (Shanghai, China).

**Table 6:** Primers used in the study

Fragment designation	Primer sequence (5’–3’)	Position	Expected size (bp)
FTY1	CgTAgA AAA AACACgggTAgAAgA	4494-4517	958
FTY2	CAggTAggTRgCACgCATATT ATT	5429-5452	
F1	ATGGGCTCCAAACCTTCTA	4550-4568	1662
F2	TCCTGTGGTGGCTCTCAT	6195-6212	
HN1	TAGAACGGTCAGAGGAGCCA	6332-6351	2095
HN2	ATGATCTGGTGCTCTGCCCTT	8407-8427	

### Sequence analysis

Nucleotide sequence editing, analysis and prediction of the amino acid sequences for both the F and HN proteins and alignments were conducted using the Clustal W multiple alignment method in the MegAlign program of the Lasergene package (DNASTAR Inc., Madison, WI, USA). Phylogenetic trees based on the F and HN genes were constructed using the MEGA 5 program (Version 5.2) with the maximum likelihood method algorithm [22]. In addition to the 55 strains collected in this study, 31 previously reported F and HN gene sequences representing different genotypes were included for comparison, and the accession numbers of each of these NDVs are shown in the phylogenetic trees [7, 16, 21].

### Pathogenicity tests

The intracerebral pathogenicity index in 1-day-old chicks and the mean death time in 9 to 11-day-old SPF chicken embryos were determined for some of the isolates in this study as Liu did previously [7].

### Cross hemagglutination inhibition (HI) test and virus neutralization test

To measure the antigenic difference between the vaccine strain LaSota and the isolated strains, we selected ten strains (One harboring 347G, four harboring 347E, and five harboring 347 K) and performed cross HI and virus neutralization test as describe by Cho and Li [8, 23]. Briefly, virus-serum mixtures were inoculated into the allantoic cavity of 10-day-old SPF chicken embryos 0.2 mL per sample. At the same time, blank controls and no-serum control samples were prepared by inoculating with an equal volume of PBS or virus. Eight days after incubation, the virus present in the chorioallantoic sac was titrated by calculating the 50 % virus neutralization endpoint. Virus neutralizing activity was determined relative to the no-serum control. The antigenic relatedness of the predominant strains and LaSota strain was expressed using the R value, as described by Archetti and Horsfall [24]. The following formula was used: $\mathrm{R}\kern0.5em =\kern0.5em \sqrt{r1*r2}$, where r1 is the titer of strain A with antiserum B divided by the titer of strain A with antiserum A, and r2 is the titer of strain B with antiserum A divided by the titer of strain B with antiserum B. A result of 0.67 ≤ *R ≤* 1.5 indicates no significant antigenic difference between the two viruses, whereas 0.5 ≤ *R* ≤ 0.67 indicates a minor antigenic difference between the two viruses. An R value of R < 0.5 indicates a major antigenic difference between the two virus strains.

### Test of the protective efficacy of the LaSota strain against the variant strains

To determine whether the vaccine strain LaSota with a low antibody level can fully protect against the variant strains, two strains, namely, JS-22-11-Ch (347E, 362G) and JS-14-12-Ch (347 K, 362A), were selected, and a locally produced, inactivated, oil-emulsion vaccine derived from the LaSota strain (10^9.3^EID_50_) was used in this study [25]. Twenty-five birds were immunized via the intramuscular route with a single dose (0.1 ml) of the LaSota vaccine. Fifteen birds were not vaccinated and served as a control group. Three weeks post-vaccination, 20 vaccinated birds were chosen and divided into two groups of 10. The birds in the La-JS22 group were challenged with 10^6.0^ EID_50_/100 μl JS-22-11-Ch (*n* = 10) via the eye drop and intranasal route (ED/IN route; 100 μl), and those in the La-JS14 group were challenged with JS-14-12-Ch (*n* = 10) via same (100 μl) route (ED/IN route). The unvaccinated birds were also challenged with JS-22-11-Ch (*n* = 5) or JS-14-12-Ch (*n* = 5) as described above and were grouped in the Control-JS22 and Control-JS14 groups. Ten birds in the Control-La and Control-PBS groups (five LaSota-vaccinated birds and five unvaccinated birds) were challenged with the same volume (100 μl) of PBS via the same route. All of the birds were monitored daily for overt clinical signs (depression, respiratory signs, diarrhea, etc.) and mortality. Tracheal and cloacal swabs were collected from birds at days 3, 5, and 7 post-challenge (pc) for virus isolation.

## Abbreviations

CEF, chicken embryo fibroblasts; F, fusion protein; HI, hemagglutination inhibition; HN, hemagglutinin–neuraminidase; L, large polymerase protein; M, matrix protein; ND, Newcastle disease; NDV, Newcastle disease virus; NP, nucleocapsid protein; P, phosphoprotein; SPF, specific-pathogen-free
